# Antiretroviral Therapy–associated Coccidioidal Meningitis

**DOI:** 10.3201/eid1901.120889

**Published:** 2013-01

**Authors:** Ronald Trible, Neil Edgerton, Salim Hayek, Daniel Winkel, Albert M. Anderson

**Affiliations:** Author affiliation: Emory University School of Medicine, Atlanta, GA, USA

**Keywords:** coocidioidomycosis, meningitis, atypical lymphocytes, IRIS, immune reconstitution inflammatory syndrome, HIV/AIDS and other retroviruses, CD4+ T-cells, antiretroviral therapy, fungi

**To the Editor:** Coccidioidomycosis, a reemerging fungal infection in the United States, comprises ≈150,000 cases annually (*1*,*2*). We report a case of postmortem examination–proven antiretroviral therapy (ART)–associated coccidioidomycosis manifesting as atypical lymphocytic meningitis, which we believe represents a rare presentation of immune reconstitution inflammatory syndrome (IRIS).

In September 2011, a 59-year-old man sought care in Atlanta, Georgia, USA, with new-onset headache, photophobia, and neck stiffness. He also reported fevers, chills, weight loss, dyspnea, and cough with scant hemoptysis. Two months earlier, he had sought care for epididymitis; HIV infection was diagnosed at that time (CD4+ T-cell count of 45 cells/µL [7%] and plasma HIV RNA level of 420,720 copies/mL [reference not detectable]). He was started on an ART regimen, and 1 week before his September 2011 illness, HIV RNA level had decreased to 790 copies/mL and CD4+ count had risen to 163 cells/µL (13%).

The patient was a thin African-American man who reported marked discomfort, with nuchal rigidity. Laboratory results were unremarkable except for serum sodium of 128 mEq/L (reference 132–144 mEq/L) and creatinine of 1.7 mg/dL (reference 0.7–1.2 mg/dL). Chest imaging showed a diffuse infiltrate in a miliary pattern. Noncontrast computed tomography scan of the head was normal. Cerebrospinal fluid (CSF) examination revealed an opening pressure of 31 cm H_2_O, 365 leukocytes/µL (reference <11/μL), (93% lymphocytes, 80% described as atypical), glucose 13 mg/dL (reference 40–70 mg/dL), and protein 171 mg/dL (reference 15–45 mg/dL).

Closer examination of the atypical CSF lymphocytes showed mostly CD3+ T cells, markedly variable in size and morphology, containing substantially irregular nuclear membranes, coarsened chromatin with multilobulated flower-shaped nuclei, and increased cytoplasm (Figure, panel A). India ink stain, acid-fast bacillus stain, and Gram stain were all negative. CSF Venereal Disease Research Laboratory and cryptococcal antigen test results also were negative. Flow cytometry on the CSF was not performed. No abnormal-appearing lymphocytes were noted in the blood.

**Figure:**
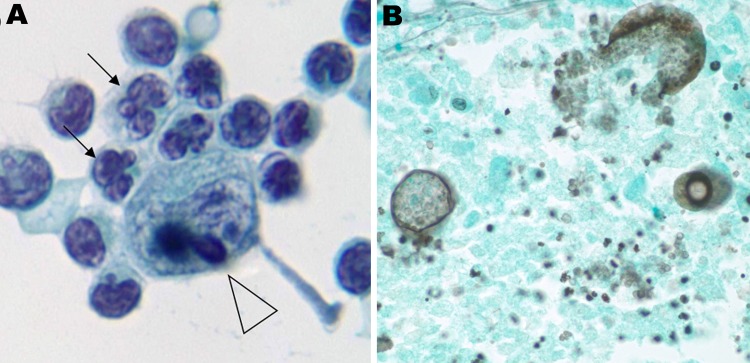
Test results of cerebrospinal fluid and brain tissue in HIV-positive man with signs and symptoms of meningitis, Atlanta, Georgia, USA, 2011. A) Atypical lymphocytes containing flower-shaped nuclei (arrows) and coarse chromocenters (arrowhead). Cerebrospinal fluid processed by Cytospin centrifuge (Thermo Electron Corp., Waltham, MA, USA) and prepared by Papanicolaou staining. Original magnification ×100. B) Gomori methenamine silver stain of sampled brain tissue showing multiple spherules and endospores of *Coccidioides* spp. Original magnification ×40.

Empirically, the patient was placed on 4-drug therapy plus dexamethasone for tuberculous meningitis, along with bacterial and viral meningitis coverage. Results of CSF cultures and viral PCR studies were negative. Magnetic resonance imaging of the brain showed 2 areas consistent with cavernous malformations but no abnormal meningeal enhancement. On day 5 of admission, blurred vision and confusion developed and rapidly progressed to obtundation. A repeat head computed tomography scan showed marked edema and transtentorial herniation, and a ventricular drain was placed. Full-strength voriconazole was added empirically for coverage of common fungal organisms. However, the patient’s clinical status worsened, and he died 2 days later.

On postmortem examination, brain pathology showed areas of necrosis, along with massive hemorrhage. Special staining of the necrotic tissue revealed marked inflammation surrounding multiple characteristic *Coccidioides* spp. spherules, 1 of which had ruptured and was spilling endospores (Figure, panel B). Examination of the lungs showed multiple granulomas that also contained coccidioidal spherules.

Pathogenic *Coccidioides* spp. is not indigenous to the Atlanta area; however, the patient was homeless and could have traveled to *Coccidioides* spp.–endemic areas. The need for increased suspicion for coccidioidomycosis in areas to which it is not endemic was highlighted further by a recent report identifying a case in Rome, Italy (*3*).

Coccidioidal meningitis is the most severe complication of coccidioidomycosis and results from lymphohematogenous spread from the lung, manifesting as fever, headache, changes in sensorium, malaise, and meningismus. CSF studies typically show 100–500 leukocytes/µL (predominantly lymphocytes), low glucose, and protein >150 mg/dL. Culture of *Coccidioides* spp. from the CSF is diagnostic but is much less sensitive than detection of anticoccidioidal antibodies in the CSF (*4*).

The most intriguing aspect of this case is the atypical pleocytosis, which initially suggested lymphomatous or tuberculous meningitis and obscured the true diagnosis of coccidioidal disease. Atypical reactive CSF lymphocytes were described in a lymphoma patient in whom coexistent cryptococcal meningitis was diagnosed (*5*). Those cells were initially confused for CNS lymphoma and caused a similar diagnostic and therapeutic dilemma.

Haddow et al. recently proposed case definitions for such clinical phenomena involving cryptococcosis, offering that newly defined cryptococcal disease identified after ART initiation be termed ART-associated cryptococcosis, with a more virulent subset of disease attributed to the unmasking of cryptococcal IRIS (*6*). We propose that the case described here parallels that described by Haddow et al. and illustrates ART-associated coccidioidomycosis. Furthermore, because of the significant inflammatory process, granulomatous pathology, and exaggerated clinical deterioration in the setting of rapid ART-induced CD4+ T-cell recovery, we suggest that this case meets the proposed criteria for the unmasking form of IRIS (*6*,*7*).

The high percentage of atypical lymphocytes described here is unusual for an IRIS response and might reflect an unusual variation of the diverse immune mechanisms used during an IRIS phenomenon (*8*). Another unusual case of coccidioidal IRIS manifested as superior vena cava syndrome (*9*). Additionally, the use of high-dose corticosteroids in the absence of antifungal therapy might have contributed to more aggressive disease progression. We suggest that the discovery of atypical lymphocytic meningitis in a patient shortly after ART-associated immune recovery should alert the clinician to the possibility of coccidioidal meningitis.
